# Spectrally Tunable Sources for Advanced Radiometric Applications

**DOI:** 10.6028/jres.111.030

**Published:** 2006-10-01

**Authors:** S. W. Brown, J. P. Rice, J. E. Neira, B. C. Johnson, J. D. Jackson

**Affiliations:** Optical Technology Division, National Institute of Standards and Technology, Gaithersburg, MD 20899; Brigham Young University, Provo, UT

**Keywords:** calibration, colorimetry, photometry, radiometry, source, tunable source

## Abstract

A common radiometric platform for the development of application-specific metrics to quantify the performance of sensors and systems is described. Using this platform, sensor and system performance may be quantified in terms of the accuracy of measurements of standardized sets of source distributions. The prototype platform consists of spectrally programmable light sources that can generate complex spectral distributions in the ultraviolet, visible and short-wave infrared regions for radiometric, photometric and colorimetric applications. In essence, the programmable spectral source is a radiometric platform for advanced instrument characterization and calibration that can also serve as a basis for algorithm testing and instrument comparison.

## 1. Introduction

Current radiometric source standards disseminated by national metrology institutes (NMIs) such as the National Institute of Standards and Technology (NIST) in the United States utilize traditional technologies, e.g., tungsten filament lamps and blackbodies; reflectance plaques and integrating spheres. These standards are spatially uniform and have spectral properties that vary smoothly with wavelength. These are important characteristics with respect to the practical, economic, and consistent dissemination of standards with values that are traceable to the International System of Units (SI) with the lowest possible uncertainties. These sources are ideal for cases where customers’ applications utilize sources with similar spectral and spatial properties. However, there are many applications of critical significance where the sources have unique properties. Many types of measurement bias exist in radiometry, and these effects are exacerbated when the calibration and the target source differ in their fundamental influencing parameters such as spectral or spatial distribution, brightness, or temporal behavior. As a consequence, while the existing sources provided by NMIs are important for general calibration applications, they tend to have limited utility for advanced characterization and calibration measurements. The absence of relevant, application-specific, radiometric artifacts to evaluate the performance of instruments, or to provide an application-specific calibration source standard, limits the ability of end users to verify the measurement results, sometimes with adverse consequences. Therefore, the need exists for advanced calibration artifacts to improve measurement accuracies in a wide variety of applications, ranging from basic colorimetric characterization to remote sensing radiometry.

In general, advanced calibration artifacts include spatially as well as spectrally complex distributions. Accordingly, there are two independent components to a full calibration platform capable of generating spatially and spectral complex scenes: a spectral component and a spatial component. Thus, a fully integrated radiometric characterization and calibration facility would have the capability of generating standardized sets of spatially complex scenes with high spectral fidelity [1]. Spectrally programmable light sources are under development for use in medical endoscopes [2]; for use as standard source distributions [3, 4]; and for general use in radiometric, colorimetric, and photometric research [5, 6], while spectral scene generators have been proposed for infrared calibrators [7].

It is time-consuming, expensive, and often impractical to develop and maintain different sets of calibration artifacts for each type of calibration, or for each anticipated application of an instrument. This has motivated the concept of a unified, generalized, radiometric calibration platform, preferably using common technologies and base elements, that can operate over a wide spectral range, from the ultraviolet to the thermal infrared, with at most modest changes to the basic structure. This platform will be used to generate a wide variety of standard calibration artifacts, i.e., source spectral and spatial distributions, needed for advanced radiometric characterization and calibration of sensors. The radiometric values of the platform can be made traceable to the values of reference standards using detector-based protocols. In addition to the reduction of uncertainties that arise from measurement bias, more efficient utilization of resources can be achieved by combining individual test, characterization, and calibration procedures that are required for a thorough understanding of a sensor’s performance into procedures that utilize advanced radiometric platforms. Savings can also be achieved by utilizing these platforms as artifacts for sensor intercomparisons for applications where multiples of “identical” units are used by a wide number of customers or participants.

In this work, we describe the development of the first component of a complete radiometric platform, an Advanced Radiometric Source (ARS), capable of generating complex spectral distributions. Several example radiometric characterization and calibration applications of the technology are described.

## 2. Advanced Radiometric Sources (ARSs)

**Fig. 1:**
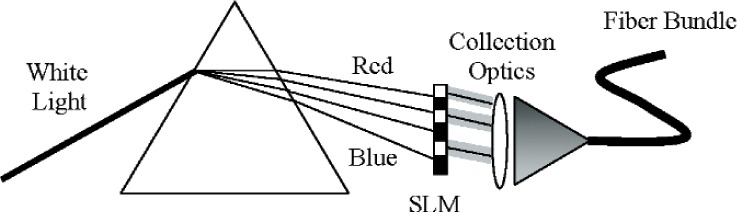
Schematic diagram of an ARS consisting of a white light source, dispersing element, spatial light modulator (SLM), collection optics, and fiber bundle.

A variety of different approaches to the development of spectrally tunable sources have recently been described, including the use of spatial light modulators [2, 4, 7, 8] or light-emitting diodes (LEDs) [3, 5, 6, 9, 10]. In this work, we describe an ARS based around a dispersing element and a spatial light modulator (SLM). The system is basically a spectroradiometer, with an SLM replacing the multi-element detector at the focal plane of the spectroradiometer (Fig. 1). In this case, the dispersed light falls on the SLM, creating a relationship between the spatial location on the modulator and the wavelength of the light. The spectral coverage is determined by the dispersing element and the size and placement of the SLM. By varying the transmittance or reflectance of the elements comprising the SLM, spectrally diverse distributions can be created. High fidelity spectral matches to a specific target distribution can be readily achieved, with the resolution defined by the design and imaging properties of the spectroradiometer.

While a variety of SLMs can be used, the two ARSs described in this work use a Digital Micromirror Device (DMD)^1^ as a spatial light modulator. DMDs are commercially available, selectively and dynamically controllable, two-dimensional, micro-mirror arrays; they are commonly found in projector displays and wide format televisions. DMDs use aluminum mirrors, and spectrally tunable sources can be created from the ultraviolet to the infrared, limited only by details of the imaging system. Replacing the window covering the DMD, the response of the SLM can be extended into the thermal infrared as well. For the ARSs described in this work, a DMD with 1024 columns × 768 rows of mirrors on a 13.68 µm pitch was used. Each mirror tilts on a hinge, and can be set to be either “on,” reflecting light to collection optics, or “off,” reflecting light to a beam dump [11]. The purpose of the collection optics is to recombine the spectrally selected output.

The fundamental radiometric distribution of the source depends on the spectral output of the primary source and the imaging properties of the spectroradiometer. An arbitrarily shaped spectrum, representing a target spectral radiance, is generated by turning on different numbers of elements of a micro-mirror column corresponding to a specific wavelength. Consequently, the source intensity can be modulated by changing the number of mirror elements “on” while maintaining a constant spectral distribution. The system can be operated in either dc or ac mode (at rates up to 5 kHz), creating either constant or time-varying spectral distributions.

Two prototype ARSs have been built, one that operates in the visible (Vis) spectral region, from approximately 400 nm to 700 nm, and one that operates in the short-wave infrared (SWIR) region, from approximately 1000 nm to 2500 nm. Common software has been developed for both platforms; the software generates a binary spectral image on the DMD that creates an arbitrary target spectrum. The target spectrum is read in from a file, peak normalized, and re-sampled to place it on the wavelength grid of the 1024 DMD image columns. Wavelength and intensity calibrations, specific to each ARS, are then applied. For the intensity calibrations, it was simply assumed that the relative intensity at a given wavelength, corresponding to a given DMD column, is proportional to the number of mirrors (from 0 to 768) turned “on” in that column. The binary images sent to the DMD are symmetric about a horizontal line drawn through its center to take advantage of the symmetry of the illumination source, which is generally aligned to be centered on the DMD.

The Vis ARS operates in the typical spectroradiometer configuration, while the SWIR ARS is configured to operate in the reverse fashion from this configuration. In the typical configuration, light goes through a slit and is dispersed before hitting the DMD. In the reverse configuration, the opposite is true. Broadband light is incident on the DMD. Reflected light from the DMD is dispersed and is then incident on a slit. Both sources are detector-based; the Vis ARS uses a spectroradiometer while the SWIR ARS has been tested with a Fourier Transform Infrared (FTIR) spectrometer. Taking advantage of the wavelength stability of spectroradiometers, both ARSs can be based on single element detectors, which is a simpler, cost-effective approach. A single-element silicon detector is suitable for the Vis ARS while either an extended Indium Gallium Arsenide (extended InGaAs) or Indium Antimonide (InSb) detector is appropriate for the SWIR ARS.

### 2.1 Prototype Visible Advanced Radiometric Source (Vis ARS)

**Fig. 2:**
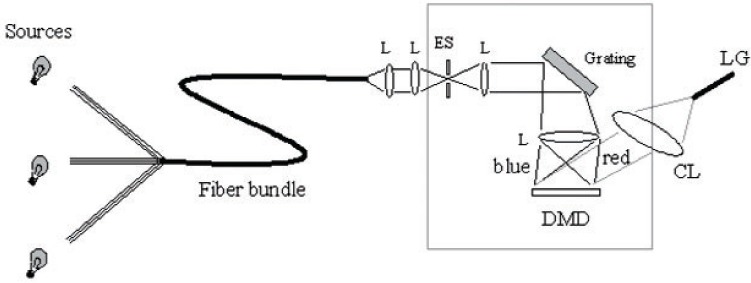
Schematic diagram of spectroradiometer-based ARS. L: lens; ES: entrance slit; CL: collection lens; LG: light guide.

The Vis ARS has the standard spectroradiometer configuration, shown in Fig. 2. Fiber-coupled incident light is collimated, then focused onto the spectroradiometer entrance slit using a cylindrical lens. Note that using multi-furcated fiber bundles enables the source distribution to be tailored for different applications. In these examples, only one 100-W Quartz-Tungsten-Halogen (QTH) lamp was used.

The incident light is collimated using a cylindrical lens, diffracted by a visible grating, then imaged onto the DMD using a second cylindrical lens. The light reflected from the DMD when the mirrors are “on” is collected by a collection lens and coupled into a liquid light guide. The light guide is introduced into a small integrating sphere, where the output is measured with a spectroradiometer. Note that the quality of any spectral match with a target light distribution is directly related to the quality of the spectroradiometer-measured spectral distribution. Any systematic errors in the spectroradiometer measurements will be incorporated into the source spectral distribution. Scattered light within a spectroradiometer is a common source of significant measurement error with these instruments. For the lowest uncertainty measurements, the spectroradiometer was first characterized and corrected for its stray light response [12].

**Fig. 3:**
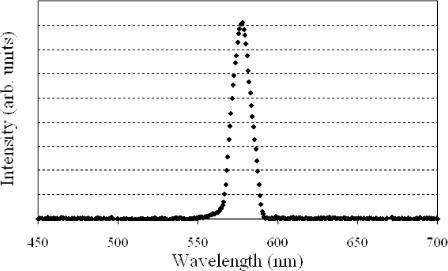
ARS distribution with 10 columns of mirrors turned “on”.

**Fig. 4:**
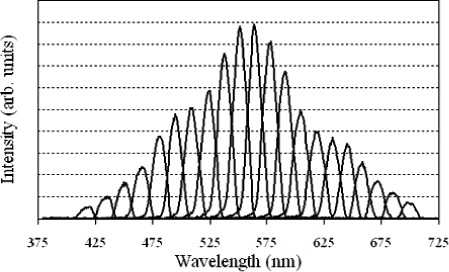
ARS distribution as bit mapped images of 10 columns of “on” mirrors across the DMD are displayed.

**Fig. 5:**
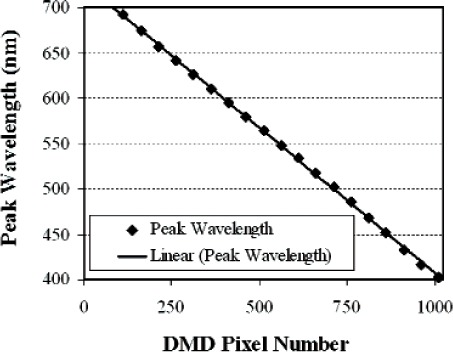
Wavelength calibration of the Vis ARS. Solid circles are measured data points; the solid line is a linear fit to the data.

A bit map image sent to the DMD consisting of a vertical strip of all elements in one or several columns 'on' corresponds to a virtual exit slit in the spectroradiometer. As different columns are turned on, the virtual exit slit moves across the dispersed image, and different spectral distributions are reflected onto the imaging mirror. Figure 3 shows the spectral distribution of the source measured by the spectroradiometer, when 10 columns are turned on. Figure 4 shows the spectral intensities and distributions as the 10-column line image moves across the DMD array. A linear relationship exists between the central DMD column turned on and the peak wavelength of the reflected light, as shown in Fig. 5. The fit to the data gives the wavelength calibration used to match different spectral distributions, while the intensity fit gives the radiant intensity calibration.

**Fig. 6:**
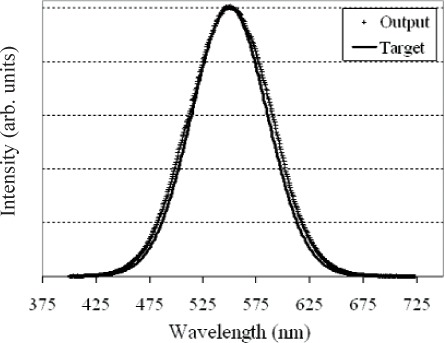
ARS match to a target Gaussian distribution. Solid line is the target spectrum; open circles are the measured ARS spectral distribution.

**Fig. 7:**
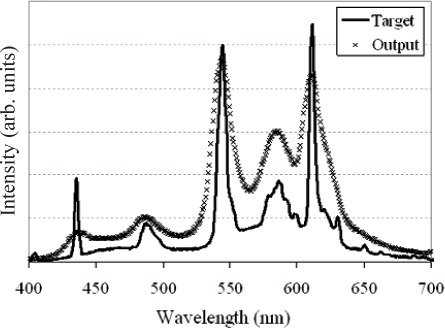
ARS match to a fluorescent lamp distribution. Solid line is the target spectrum; open circles are the measured ARS spectral distribution.

**Fig. 8:**
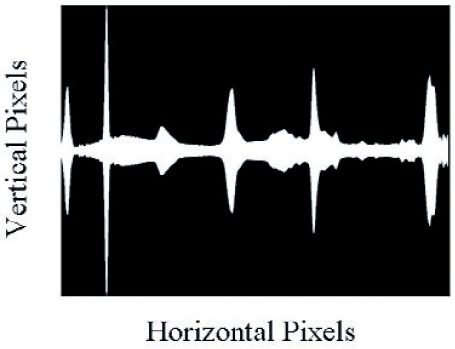
The binary spectral image written to the DMD in the SWIR spectral light engine that resulted in the measured spectrum of Fig. 7. White regions map to “on” mirrors and black regions map to “off” mirrors.

Complex spectral distributions are generated by turning on series of mirrors across the DMD array. Figures 6 and 7 show example fits to complex spectral distributions. Figure 6 corresponds to a Gaussian distribution, while Fig. 7 is the spectral distribution of a fluorescent lamp. Figure 8 shows the bit-mapped image sent to the DMD to generate Fig. 7. The white regions correspond to mirrors turned “on” while the dark regions correspond to mirrors turned “off ”.

### 2.2 Prototype Short-Wave Infrared Advanced Radiometric Source (SWIR ARS)

**Fig. 9:**
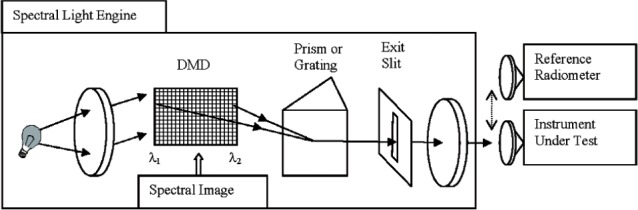
Basic scheme of the spectral light engine in reverse spectroradiometer mode.

**Fig. 10:**
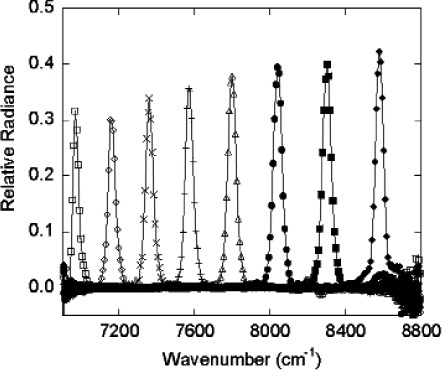
Mirror columns turned on across the DMD array. Spectra used for wavelength calibration.

**Fig. 11:**
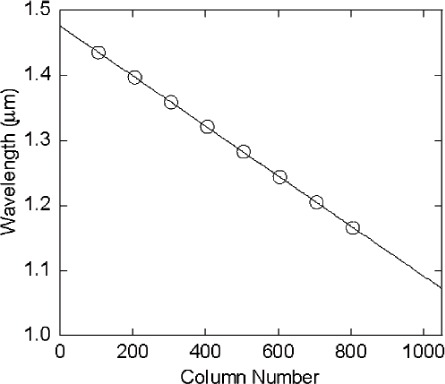
A plot of peak wavelength of the spectra shown in Fig. 10 versus the corresponding DMD strip central column number, along with the linear fit used for wavelength calibration.

For the SWIR ARS, a single-grating commercial spectroradiometer was used in reverse mode (Fig. 9, with a prism substituted for the grating as the dispersion element): the exit slit of this monochromator was removed, but its entrance slit was retained and functions as the STS exit slit. An off-axis spherical aluminum mirror imaged the light source onto the STS entrance aperture such that the DMD was just overfilled by the source. When the bit-mapped image sent to the DMD is a vertical strip, the image at the STS exit aperture (spectroradiometer entrance aperture) is that of a virtual entrance slit. Due to the spectral selectivity provided by the prism (or diffraction grating) combined with the real exit slit, the vertical slit generates a quasi-Gaussian profile, centered at a single wavelength, with a full-width half-maximum (FHWM) bandwidth of approximately 8 nm, as shown in Fig. 10. In this figure, each spectrum was taken with a 10-column wide vertical strip as the DMD image. Tuning of the wavelength is achieved by moving the horizontal position of this virtual entrance slit by simply writing a new, horizontally displaced vertical strip image to the DMD. Eight spectra are shown in Fig. 10; each spectrum corresponds to turning on a different set of columns. A plot of the peak wavelength for each spectrum versus the corresponding DMD strip central column number is shown in Fig. 11. As with the Vis ARS, there is a direct relationship, nominally linear, between each DMD column and the resulting center wavelength of the image that passes through the system’s exit slit. A linear fit of the wavelength versus column number relationship from Fig. 11 constitutes a wavelength calibration of the STS.

**Fig. 12:**
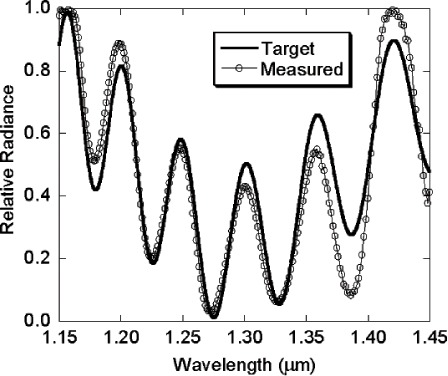
An example target spectrum (solid line with no symbols) and the resulting measured spectrum (symbols connected with solid line) from the SWIR spectral light engine.

**Fig. 13:**
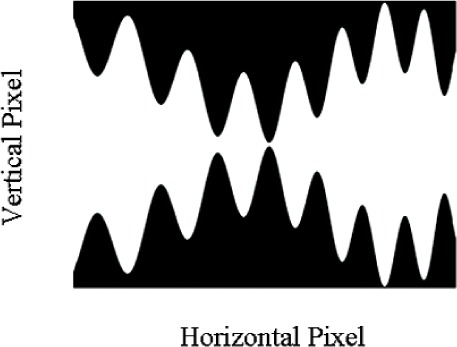
The binary spectral image written to the DMD in the SWIR spectral light engine that resulted in the measured spectrum of Fig. 12. For display of such images on the DMD, white regions map to “on” mirrors and black regions map to “off” mirrors.

Displaying the image of Fig. 13 on the DMD resulted in the measured spectrum shown in Fig. 12, which is plotted along with the original target spectra. The white regions in the image correspond to mirrors turned “on” while the dark regions correspond to mirrors that were turned “off”.

For both the Vis and the SWIR ARS, there is a good qualitative match to the wavelength and the general features of the relative intensity of the desired spectrum. These examples demonstrate the feasibility of the development of a common radiometric calibration platform covering the visible and short-wave infrared spectral regions. However, there are significant differences in the relative radiance. Note that no algorithm was developed to take account of the spectral width of a column of mirrors or for the bandpass of the spectroradiometer. In addition, no feedback was provided to iteratively adjust the displayed DMD image to generate successively better matches to the target spectra. Incorporating these two elements into the prototype STS should result in high-quality matches to complex target spectral distributions.

## 3. Example Applications of the Visible Advanced Radiometric Source

In the following, two example applications of the Vis ARS are given, one radiometric application and one colorimetric application. Note that the same ARS can be used for both applications, producing different sets of spectral distributions tailored to the specific application.

### 3.1 Example Radiometric Application

Under natural illumination from sunlight, the optical properties of seawater depend strongly on the constituents in the water. Dissolved and suspended materials give rise to spectrally dependent absorption, scattering, and fluorescence. For quantitative studies of the ocean, the optical properties are related to physical and biogeochemical data products through bio-optical algorithms. For example, due to the presence of chlorophyll-*a*, phytoplankton absorb blue light strongly and reflect predominantly green light. Therefore, the ocean color, or water-leaving radiance, can be related to the concentration of phytoplankton chlorophyll-*a*, and global ocean color measurements by satellite sensors can give information regarding the concentration and distribution of microscopic marine plants and organisms.

**Fig. 14:**
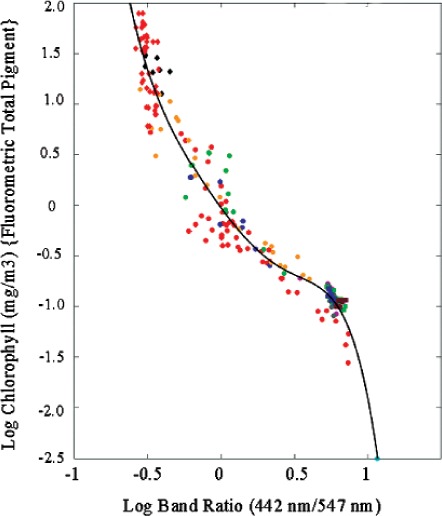
Bio-optical algorithm relating spectral band ratio to measured phytoplankton chlorophyll-*a* concentration.

Bio-optical algorithms based on the ratios of specific spectral bands relate the water-leaving radiance to physically measured biological data products. For example the ratio of 10 nm wide spectral bands centered at 442 nm and 547 nm is related to total chlorophyll-*a* according to Fig. 14 [13]. Because of the strong dependence of chlorophyll-a concentration on the measured band ratios (note the vertical axis is the logarithm of the chlorophyll-a concentration), small errors in the sensor's spectral responsivity arising from spectral shifts in filters or finite out-of-band response can cause significant errors in the bio-optical algorithm or the measured bio-optical data product.

A tunable source that can match measured or modeled in-water or above-water spectral radiance distributions would enable a new era for laboratory measurements of ocean color radiometer performance. These laboratory measurements can be used to validate the responsivity of ocean color instruments and identify underlying sources of radiometric bias. It is important to make these measurements in a laboratory setting due to the large environmental uncertainties inherent in field measurements. For instruments that have significant measurement errors, correction algorithms based on band ratios can be developed that would greatly reduce the magnitude of the errors [14].

**Fig. 15:**
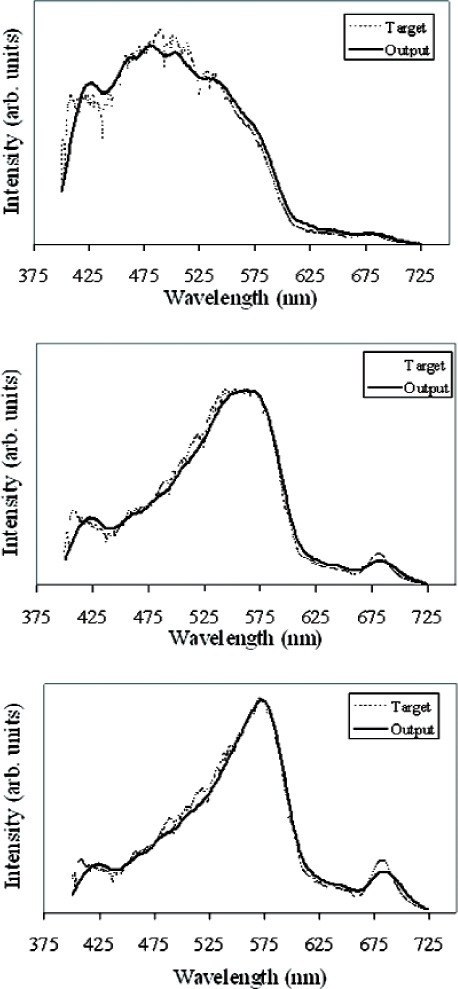
Spectral matches to measured water-leaving radiance distributions for chlorophyll-*a* concentrations of (top) 0.6 µg/l (middle) 5.65 µg/l and (bottom) 12.44 µg/l. Dashed line is the measured spectrum; solid line is the DMD-generated spectral distribution.

Figure 15 shows the ARS matches to measured water-leaving radiance with differing chlorophyll-*a* concentrations. The coarse structure of the different spectral distributions is matched extremely well. The fine structure, attributed primarily to Fraunhofer lines in the solar distribution, is not matched as well, due in part to the 10 nm bandwidth of the ARS. Note that spectroradiometers with 3 nm bandwidth (or spectral resolution) are readily commercially available, and use of these instruments in the ARS would enable a closer match of the ARS spectral distribution to the fine structure target distribution. Incorporation of an ocean-color ARS into measurement activities that are designed to produce consistent and accurate calibration and validation datasets for satellite sensors, such as the Sensor Intercomparison and Merger for Biological and Interdisciplinary Oceanic Studies (SIMBIOS) Project [15], would greatly simplify the measurement steps necessary to achieve the Project goals.

### 3.2 Example Colorimetric Application

**Fig. 16:**
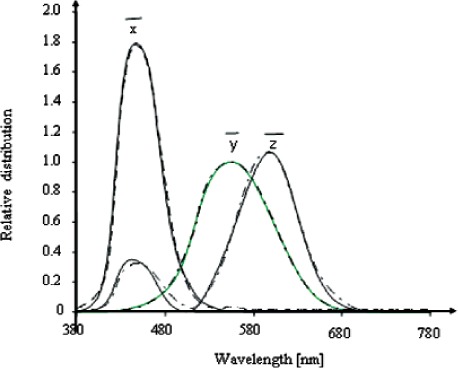
CIE-defined $\bar{x}$, $\bar{y}$, and $\bar{z}$, color-matching functions (solid lines) and RSRs of the filter channels of a colorimeter (dashed lines).

Colorimetric quantities of a light source can be measured with a colorimeter that has three or four channels consisting of detectors with spectral filters; the channels are designed to have relative spectral responsivities (RSRs) that mimic the color matching functions defined by the International Committee on Illumination (CIE) [16]. No colorimeter channel exactly matches each of the color matching functions; an example of the RSRs of colorimeter channels and the CIE-defined color matching functions is given in Fig. 16. Due to this imperfect matching of the spectral responsivities, measurement errors are inevitable. These measurement errors can increase dramatically when the relative spectral power distribution (SPD) of a test source is dissimilar to that of the calibration source. Colorimetric quantities can also be measured with a spectroradiometer, with colorimetric quantities calculated from the measured spectral power distribution [17]. Spectroradiometers theoretically do not have the spectral mismatch problem, but they are still susceptible to measurement errors due to wavelength error, stray light, and finite measurement bandwidth. These radiometric measurement errors introduce errors in calculated photometric and colorimetric quantities in a similar way that the spectral mismatch errors do in colorimeters.

Colorimeters and spectroradiometers are normally calibrated against an incandescent standard lamp operated at a correlated color temperature of ≈2856 K, approximating the CIE Standard Illuminant A [18]. When the instruments subsequently measure common colored sources, e.g., displays, fluorescent lamps, colored tiles, etc., measurement errors can be significant. For example, inter-instrument variations for chromaticity measurements of various colors of a display are as large as 0.01 in chromaticity coordinates (*x, y*) and 10 % in luminance (*Y*) while the manufacturers’ specifications (specified for Illuminant A) are 0.002 in *x, y* and 2 % in *Y* [19]. Higher accuracies are needed in many applications; for example, international standards require a measurement uncertainty of 0.005 or less for chromaticity and 4 % or less for luminance when measuring red, green, blue and white colors of displays [17, 20].

Using the ARS, a variety of spectral distributions can be readily generated to provide a more useful or accurate metric for the evaluation of the performance of colorimeters for both general and specific applications. In one specific application, a spectrally tunable source, capable of matching the spectral distribution of primary red, green, blue and white colors of displays, would enable accurate and rapid calibration of a colorimeter or spectroradiometer used to measure the colorimetric properties of the display [5].

In a more general application, the source can be used to generate spectral distributions according to established protocols that evaluate the performance of colorimeters. For example, protocols can be developed to generate a colorimeter Color Measuring Index (CMI), which can be thought of as the detector analog to a source Color Rendering Index (CRI) [21]. The CRI is a way of measuring how well a given light source can reproduce colors in comparison to a reference light source (e.g., daylight). To generate the CRI for a source, the tristimulus values of a number of test color samples under both the illuminant to be tested and a reference illuminant are calculated knowing the relative spectral power distribution of the source. Numerical values are given, based on the color differences in object color space between the test and reference sources. The average of these numbers gives the CRI. There are a total of 14 test color samples with reflectances defined by the CIE [21]. A subset of eight test-color samples that cover the hue circle, are moderate in saturation, and are approximately the same in lightness, is used to generate the general color rendering index R_a_. An additional set of six color samples are often included representing a strong red, yellow, green, and blue; and representing complexion and foliage colors.

To generate a colorimeter CMI, a set of spectral distributions can be generated by the ARS, and their colors measured by the colorimeter and a reference spectroradiometer. Then, the differences between the colors measured by the colorimeter and the reference spectroradiometer can be calculated, and a numerical value assigned for each spectral distribution based on the measured difference. A general CMI would be the average of the numerical values for the measured spectral distributions. The set of spectral distributions generated by the ARS will be determined by a standards organization, but could in principle be the same or similar to the distributions used to generate a CRI for sources. In this application, the RSRs of the colorimeter channels do not need to be known, and the CMI is based on measured, not calculated, values. The availability of the ARS enables source distributions to be realized that are no longer to be based on material samples (i.e., colored ceramic tiles) and Planckian excitation source spectral distributions. This gives standards organizations a great deal of flexibility in the defined spectral distributions to be measured not previously available, and should result in a useful, practical metric describing the general performance of the colorimeter.

## 4. Conclusions

With the trend toward performance-based metrology requirements, NMI’s well-defined role in establishing and defining metrics to verify the performance of radiometric systems is becoming more critical. Current calibration artifacts lack the spectral and spatial complexity often required for advanced radiometric calibration protocols necessary to achieve the requisite instrument performance for a defined application. Cost effective artifacts and efficient means of scale dissemination must be achieved. The development of spectrally tunable sources enables NMI measurement and calibration services to respond to new radiometric requirements by providing necessary, relevant technology that tests the end performance of radiometric systems used in critical applications. The next step involves incorporating the advanced radiometric sources in high fidelity spatial scene generators to produce complex spatial scenes for the characterization of imaging systems [1]. Together, these two advanced radiometric platforms will form the backbone for advanced radiometric characterization and calibration protocols for UV, visible and infrared sensors and systems.

## Footnotes

1 Certain commercial equipment, instruments, or materials are identified in this paper to foster understanding. Such identification does not imply recommendation or endorsement by the National Institute of Standards and Technology, nor does it imply that the materials or equipment identified are necessarily the best available for the purpose.
